# Wireless sensing in high-speed railway turnouts with battery-free materials and devices

**DOI:** 10.1016/j.isci.2023.108663

**Published:** 2023-12-07

**Authors:** Yuhua Sun, Yan Yan, Song Tian, Gang Liu, Fei Wu, Ping Wang, Mingyuan Gao

**Affiliations:** 1 College of Engineering and Technology, Southwest University, Chongqing 400716, China; 2 School of Materials and Energy, Southwest University, Chongqing 400715, China; 3 College of Mechanical and Vehicle Engineering, Chongqing University, Chongqing 400030, China; 4 School of Civil Engineering, Southwest Jiaotong University, Chengdu 610031, China; 5 Key Laboratory of High-speed Railway Engineering, Ministry of Education, Chengdu 610031, China

**Keywords:** Applied sciences, Engineering, Materials science

## Abstract

Sustainable energy technologies enable solutions for future green transportation. Realizing status awareness and effective wireless monitoring of rail transit infrastructure in dark environments, narrow spaces, and unattended conditions has always been a challenge. This study presents a battery-free vibration-powered force sensing system (VFSS) that integrates structural loading, sensing, and energy harvesting. The proposed VFSS can sense a switching force of up to 4 kN in the high-speed railway turnout section, deliver 6.9 mW of average power over a broad frequency band (ca. 500 Hz) under a vibration amplitude of 0.2 mm, and realize wireless data transmission. Through a cross-scale design from the device to the system, we demonstrate an all-in-one smart component that features stress flow, signal flow, and energy flow, which could highlight the implementation of energy structures in the future.

## Highlights

• An enhanced piezoelectric energy harvester with force amplification
• Railway track vibration energy harvesting under broadband micro amplitude excitation
• An axle pin serves as both a structural fastener and a sensor
• All-in-one smart component that features stress flow, signal flow, and energy flow

## Graphical abstract

**Figure:**
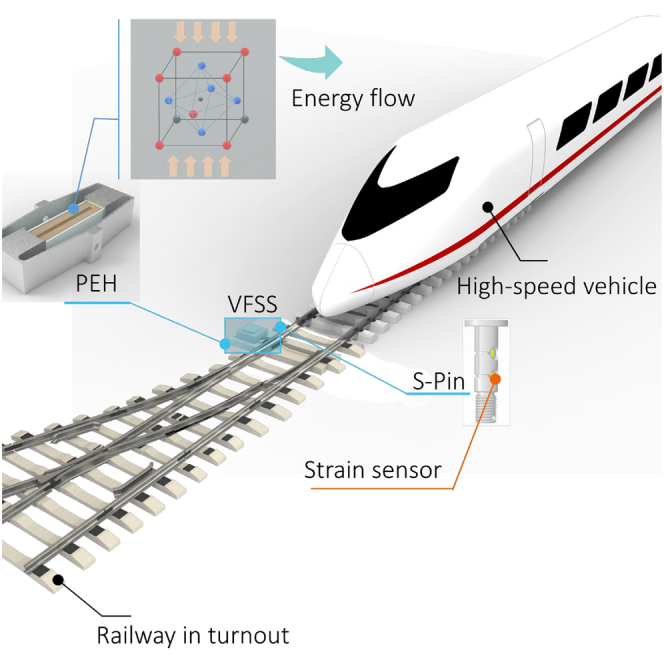

## Introduction

### Research motivation

There is a global consensus to develop low-carbon, renewable, and clean energy.^1^^,^^2^ In the past decade, technological progress in green energy has made several breakthroughs,^3^ particularly in the fields of classic renewable energies, such as solar power^4^^,^^5^ wind power,^6^ hydropower,^7^ geothermal energy,^8^ bioenergy,^9^ and marine energy.^10^ However, the above-mentioned renewable energy sources depend on the region, and their application is considerably restricted in areas with or without limited renewable energy resources.

In contrast, vibration energy exists widely in the environment and can serve as a promising micro-energy source; therefore, it has attracted considerable attention in recent years.^11^^,^^12^ Compared with traditional renewable energy sources, vibration energy has a low power capacity and cannot provide usable energy to the power grid. However, owing to the wide distribution of environmental vibration sources, they have broad application prospects in various applications, such as the Internet of Things (IoT) and distributed self-powered microgrids.^13^

Cutting-edge research on vibration-based energy harvesting primarily focuses on new materials and fabrication processes for piezoelectric and triboelectric nanogenerators. Moreover, various innovative structures using nonlinear mechanisms have been reported. However, it is still challenging to develop autonomous battery-free sustainable monitoring nodes at the system level by comprehensively coordinating the device design, energy management, electronic circuits, and system integration.

As a type of high-capacity transportation, rail transit has developed rapidly worldwide in recent years. The turnout system is a “steering wheel” to ensure that trains run safely in a selected route. A switch machine or turnout motor is the core component of a turnout system for controlling the alignment of the turnout and realizing the steering of trains.^14^ As the operational stability of switch machines directly affects the efficiency and safety of railway systems, it is essential to monitor the status of switch machines in real-time.^15^

**Figure 1:**
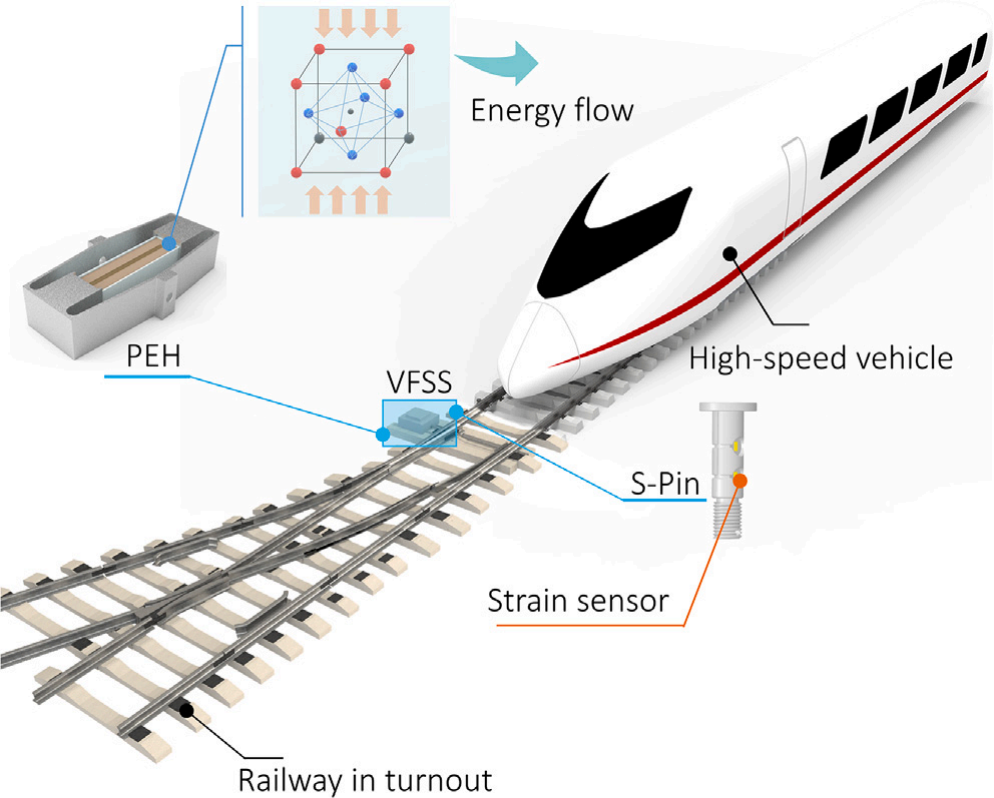
VFSS in high-speed railway turnout by piezoelectric energy harvesting and strain sensor.

Herein, we present a VFSS for high-speed railway turnout using piezoelectric energy harvesting and strain sensors (as illustrated in Figure 1). The proposed system has the capacity to self-supply energy and perform real-time monitoring of the switch machine, which can effectively reduce the safety risks caused by undiscovered component defects. The ceramic perovskite material-lead zirconate titanate (PZT), with the chemical formula Pb[Zr0.52Ti0.48]O3, was synthesized and prepared in the form of a film. A plurality of PZT thin-film units was stacked, and the electrodes were bonded according to the polarization direction and connected in series and parallel to form a block-shaped piezoelectric transducer. Once the PZT transducer block is embedded in a metal spring jacket (MSJ) with a force amplification effect, a significant amount of electricity can be generated under the action of external vibration acceleration. The collected electrical energy was used to power the strain sensor. The strain film sensors were integrated into the bearing shaft pin, and the tensile and compressive forces on the shaft pin could be sensed to provide real-time data for safe operation and continuous monitoring of railway switch machine systems.

### Literature review

The railway turnout system is a vital piece of infrastructure that is in charge of directing trains. The degree of proximity of the turnout is closely connected to the safety of passing trains. To avoid derailment incidents caused by insufficient turnout closeness degrees, turnout closeness degrees must be monitored.^16^ Every year, a significant number of derailment events and turnouts are recorded across the world. These mishaps not only entail operational delay and financial damage, but they can also result in injuries and, in some cases, fatalities.^17^ Nowadays, a variety of condition-monitoring strategies (e.g., video perception technologies, machine learning algorithms, efficient fault predictions, advanced measurements, and energy harvesting principles.) are proposed to detect a turnout’s working performance.

Real-time data of service status indicators were acquired using fiber grating and video perception technologies from the perspectives of video perception technologies. A real-time monitoring system for the service status of the bridge’s continuously welded turnouts has been in place for more than four years, effectively maintaining railway safety and security^18^ A computer vision approach for detecting wear on railway automated switch stationary contacts was presented, which computes average pixel distance in wear zones and rescales to real-world level using image calibration tools.^19^ Furthermore, an image sensor-based detector was constructed, and an automated technique was presented to assess the size of the switch gap using the detector’s pictures.^16^^,^^20^ The picture feature recognition technique was created to enhance the monitoring and maintenance of high-speed railway turnout gaps in order to minimize the substantial impact of train operation on the turnout.^21^

From the standpoint of a machine learning algorithm, to accomplish intelligent detection of turnout defects against the backdrop of big data,^22^ an intelligent diagnostic technique based on deep learning curve segmentation and the Support Vector Machine was provided, and the suggested method’s diagnostic accuracy can reach 98.5%.^23^ In addition, an intelligent diagnosis approach for railway turnouts using Dynamic Time Warping was created to analyze five different types of turnout faults. According to the analytical results, it could be diagnosed automatically with 100% accuracy.^24^ Furthermore, an online diagnosis system based on Bayesian incremental learning and scalable fault identification^25^ and a semi-supervised learning algorithm-based technique for detecting aberrant states of the turnout action curve,^26^ displaying excellent precision, flexibility, and efficiency, was proposed to monitor the operation status of turnouts.

In order to focus on the jamming problem caused by inadequate lubrication in railway turnout systems, an unsupervised adaptive latent feature extraction approach based on the enhanced sparse auto-encoder was given.^27^ An approach for identifying possible fault occurrences of railway point-operating devices using unlabeled signal sensor data was developed.^28^ The sectionalized feature extraction technique of the electrohydraulic switch machine’s oil pressure signal^29^ and the turnout system’s optimal operation with maintenance^30^ were depicted in order to perform fault diagnosis and forecast the state of the electrohydraulic switch machine. Furthermore, to improve overall fault-detection performance, a Kalman filter for the linear discrete data filtering problem encountered when using current sensor data in a point condition monitoring system was proposed to predict problems and enable quick recovery before component failures disrupt operations.^31^^,^^32^ It is critical to automate the monitoring of turnouts using fault-detection algorithms.^33^ As a result, an unsupervised fault-detection approach based on deep auto-encoders was devised, consisting of an unknown mode mining stage and a multimode fault-detection stage.^34^

Various suitable condition monitoring techniques, to deal with fault detection and diagnosis in railway switch and crossing systems,^35^^,^^36^ e.g., various prognostic methods,^37^ measurements by parameters from a track recording car,^38^^,^^39^ ways for determining the correct geometric center of a turnout -monitoring and inspecting the geometric center of a double and outer slip turnout,^40^ the electronic analysis system of crossing system for railway turnouts' inertial measurement technique,^41^ has enabled academics, railway operators, and professionals to investigate, develop, and implement the finest ways for their applications. Mobile laser scanning,^42^ adherence detector,^43^ and measured sleeper accelerations and scanned crossing geometries from six *in situ* crossing panels^44^ were also used to continuously check the status of railway crossings and switches. Moreover, direct techniques for simultaneously monitoring switching time, switching power, voltage, current, and so forth were also described.^45^^,^^46^

Moreover, an innovative solution for monitoring the status of temperature and other atmospheric conditions, such as snow accumulation in turnout areas was presented to monitor the working condition of railway turnouts.^47^^,^^48^ The real-time damage evaluation of the switch condition index was designed to standardize the inspection process. It was based on the findings of the field study, statistical analyses of engineering experiences, and expert opinions utilizing the Delphi approach.^49^^,^^50^ A hybrid wind energy harvesting system based on self-adapting drag-lift conversion was created to power high-speed railway turnout monitoring from the standpoint of the self-powered monitoring system for the operational performance of the switching machines^51^ and a self-powered rail health monitoring system^52^ and a turnout monitoring system were proposed based on the triboelectric nanogenerator.^53^

Although the aforementioned methods have high accuracy and robustness, which simplifies the turnout closeness maintenance process, a large amount of data needs to be processed. High requirements of hardware are essential in these application scenarios. Furthermore, special sensors and complex signal processing programs are requisite in most of the adopted methods. The most crucial point is that external power is essential for the power supply. Herein, we consider the switching force of the switch machine in a high-speed railway turnout section as the monitoring object to present an unattended battery-free sensing and wireless monitoring node from materials to the device and system.

### Scope of article

As shown in Figure 1, the unattended battery-free sensing and wireless monitoring node has the profile of an axle pin, which itself is both a structural fastener and a strain sensor as sensing elements. It is powered by a piezoelectric energy harvester (PEH) stacked layer-by-layer with Pb[Zr,Ti]O_3_ (PZT) ceramic plates and a power management (PM) unit for AC–DC conversion, charge pumping, boost control, charge storage, and distribution. We demonstrate that the proposed node can sense a switching force of up to 4 kN in the high-speed railway turnout section, deliver an average power of 6.9 mW over a broad frequency band (ca. 500 Hz) under a small vibration amplitude of 0.2 mm, and realize wireless data transmission. We exhibit an all-in-one smart component that features stress, signal, and energy flows in a cross-scale design from materials to the device to the system, which may shed light on the implementation of future energy structures.

Currently, the evaluation of the connecting rod force in switch machines predominantly depends on manual periodic inspections. To conduct these inspections, professionals begin by removing the fixing bolt and then placing a force sensor in the bolt’s original location. The station dispatcher is responsible for operating the switch rail, and activating and deactivating it as needed. During this time, the inspection staff monitors the switch machine’s condition by analyzing force data displayed on their portable digital devices. This manual approach is characterized by its low efficiency and high inspection costs, requiring implementation during specific operational downtimes.

In contrast, the method proposed in this article offers several advantages over the traditional technique. It presents the potential to significantly reduce costs and improve efficiency, while also enabling the implementation of online, real-time force monitoring. This advanced approach represents a substantial improvement in the field of switch machine maintenance and operation.

This research presents an innovative solution aimed at addressing the existing challenges associated with fault detection and diagnostics in railway switch and crossing systems. Specifically, an unattended battery-free sensing and wireless monitoring node for switch machine performance has been developed to bridge this gap. The study offers several unique aspects and contributions, as outlined later in discussion: (1) Introduction of an enhanced piezoelectric energy harvester with force amplification: This innovation effectively converts vertical vibration acceleration into longitudinal force, thereby driving the piezoelectric device to transduce energy. (2) Development of track vibration energy harvesting under broadband (approximately 500 Hz) micro-amplitude (0.2 mm) excitation: This advancement allows for efficient energy harvesting from track vibrations. (3) Design of an axle pin with dual functionality as a structural fastener and a sensor: The installation process for this axle pin is highly convenient, and it demonstrates a high level of accuracy in target detection. Furthermore, it possesses the capability to accurately assess strain levels in high-speed railway turnouts, even in diverse and challenging environmental conditions. (4) Validation of the feasibility of an all-in-one smart component: This component integrates stress flow, signal flow, and energy flow. The primary role of the axle pin strain sensor is to quantify the force exerted by the connecting rod of the switch machine. This information is wirelessly transmitted through a dedicated circuit responsible for relaying the sensed force. Notably, both the sensor and the circuit are powered by a piezoelectric energy harvester. The successful integration of these functionalities into a single smart device has been demonstrated and verified.

The remainder of the essay is structured as follows: Piezoelectric energy harvester design (Including Fabrication of the piezoelectric energy harvesting block, Material characterization of the PZT ceramics, The design, simulation and dynamic response of the MSJ and Power generation capacity of the PEH) are discussed in Section design of the piezoelectric energy harvester. Detailed piezoelectric strain sensing and force magnification by axle-pin, including design of the axle pin sensor and modeling and calibration of the axle pin sensor, is described in Part 3. On-site system integration testing of the VFSS in high-speed railway turnout is illustrated in Section on-site system integration testing of the VFSS in high-speed railway turnout. The conclusion is included in Part 5.

### Design of the piezoelectric energy harvester

#### Fabrication of the piezoelectric energy harvesting block

PZT ceramic samples were fabricated using a conventional die-pressing and sintering process. High-purity commercial PZT powder (Baoding Chemicals, China) was mixed with 8-weight percent polyvinyl alcohol (PVA) gel. Following granulation, the samples were subjected to a pressure of 30 MPa while being pressed into discs with a thickness of 1 mm and diameter of 10 mm. The resulting green PZT discs were densified in a muffle furnace at 1200°C for 2 h.

**Figure 2:**
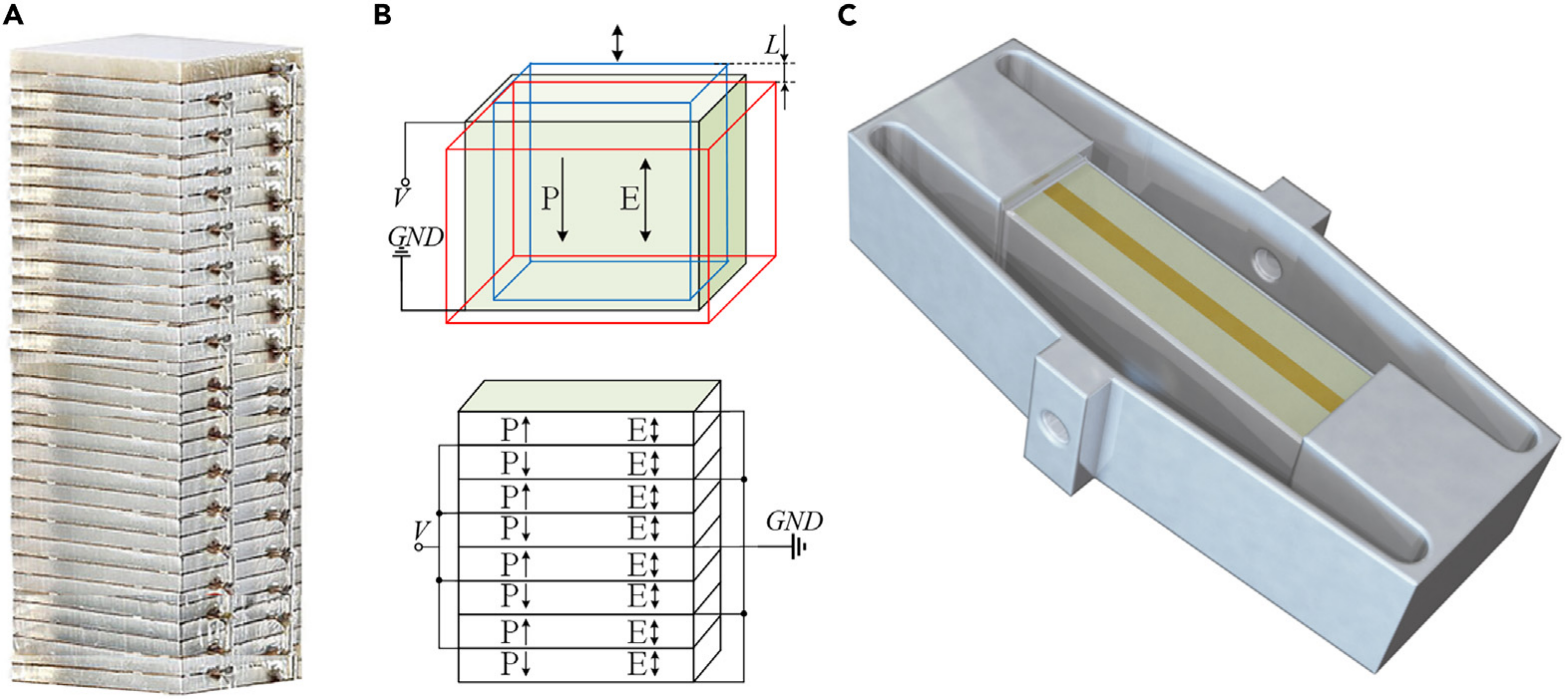
Illustration of the PEH (A) Stacked piezoelectric energy harvesting block by PZT films. (B) Polarization direction and electrical connection. (C) PEH embedded in the metal spring jacket (MSJ).

The PZT piezoelectric ceramics were stacked in pairs according to the back-to-back polarisation direction via the stacking process as shown in Figure 2. The conversion process from mechanical energy to electrical energy of the piezoelectric stacked harvester induces a change in the electrical polarization of the piezoelectric crystal when the external pressure acts, thereby generating electricity. However, before applying to the railway scenario, there are two problems to be solved:1) how to convert the vibration energy of the railway track into force on the surface of the piezoelectric device. 2) The maximum travel range of the piezoelectric stack structure is several micrometres, which is insufficient to generate sufficient power to drive the sensors; therefore, a strain amplification mechanism is required.

### Material characterization of the perovskite material-lead zirconate titanate ceramics

**Figure 3:**
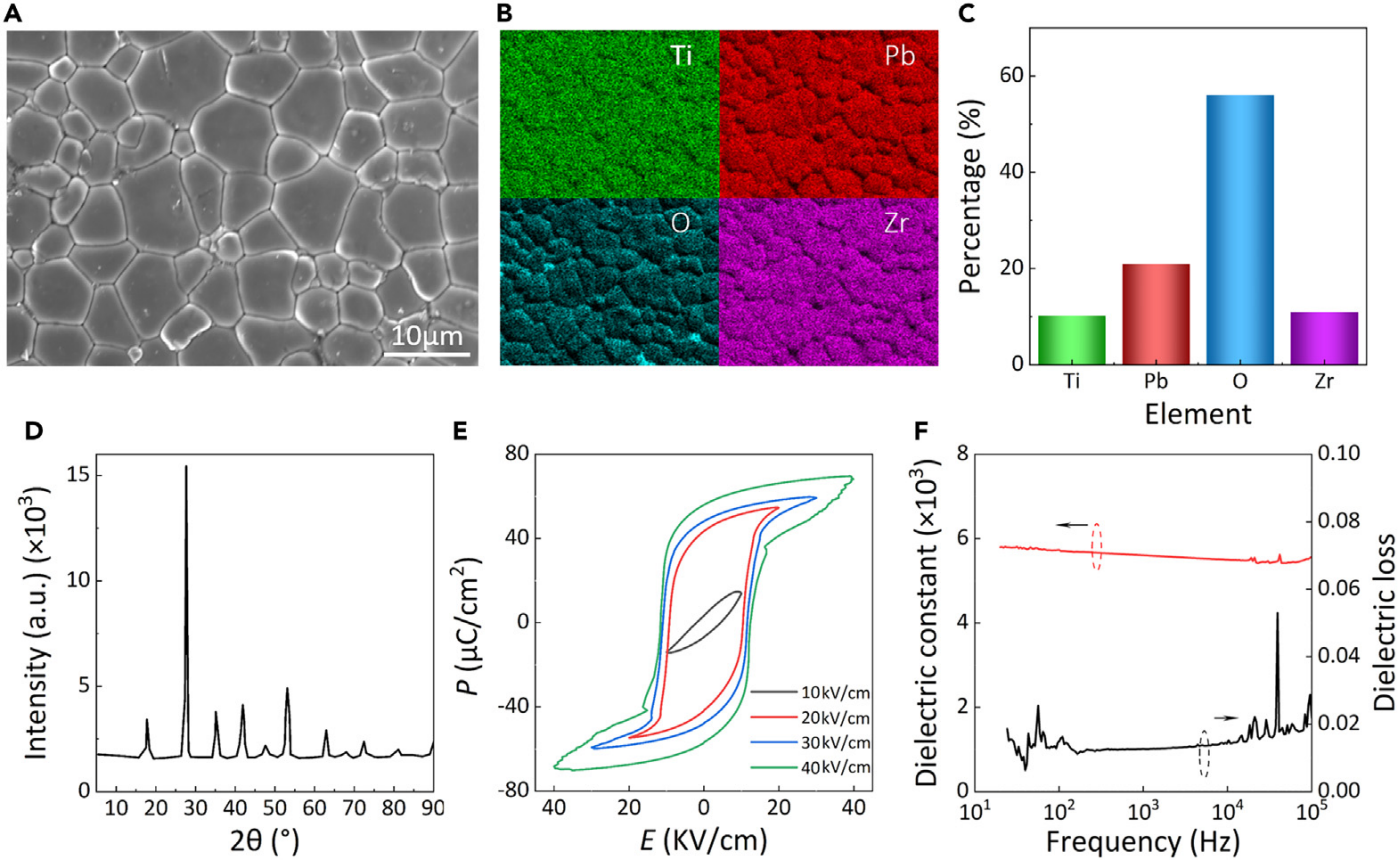
Piezoelectric energy harvesting from railway vibration by PZT and energy stack structure (A) Scanning electron microscopy (SEM). (B) Energy-dispersive X-ray spectroscopy (EDS) mapping of the lead zirconate titanate (PZT) sample (scale bar: 10 μm). (C) Elemental composition of the PZT samples. (D) X-ray diffraction (XRD) of the PZT sample. (E) The P-E curves of the PZT sample under different electric fields *E*. (F) Dielectric properties of the PZT sample at a frequency from 100 Hz to 100 kHz.

The microstructure and elemental mapping of the PZT ceramics are illustrated in Figures 3A and 3B, respectively. The PZT ceramic exhibited a dense structure with relatively homogeneous grain sizes, and the distribution of elements was uniform. The proportions of Pb, Zr, Ti, and O are shown in Figure 3C. The X-ray diffraction (XRD) spectrum indicates that the PZT ceramic had a typical perovskite structure (Figure 3D). The hysteresis loops of the PZT ceramics at different *E* values at 25°C and 10 Hz are presented in Figure 3E. The maximum and residual polarisations were approximately 67 μC/cm^2^ and 58 μC/cm^2^, respectively, under an electric field of 40 kV/cm. The relative dielectric permittivity (*ε*_*r*_) and loss tangent (tan *δ*) at frequencies from 100 Hz to 100 kHz are illustrated in Figure 3F. The dielectric constant was maintained above 5000 over a wide frequency range.

### Theoretical model and dynamic response

#### Derivation of the force amplification ratio

This part develops an analytical model to forecast the force amplifier’s force amplification ratio. The theoretical model for the frame serving as a force amplifier was created by referring to the theoretical model in the literature.^54^

**Figure 4:**
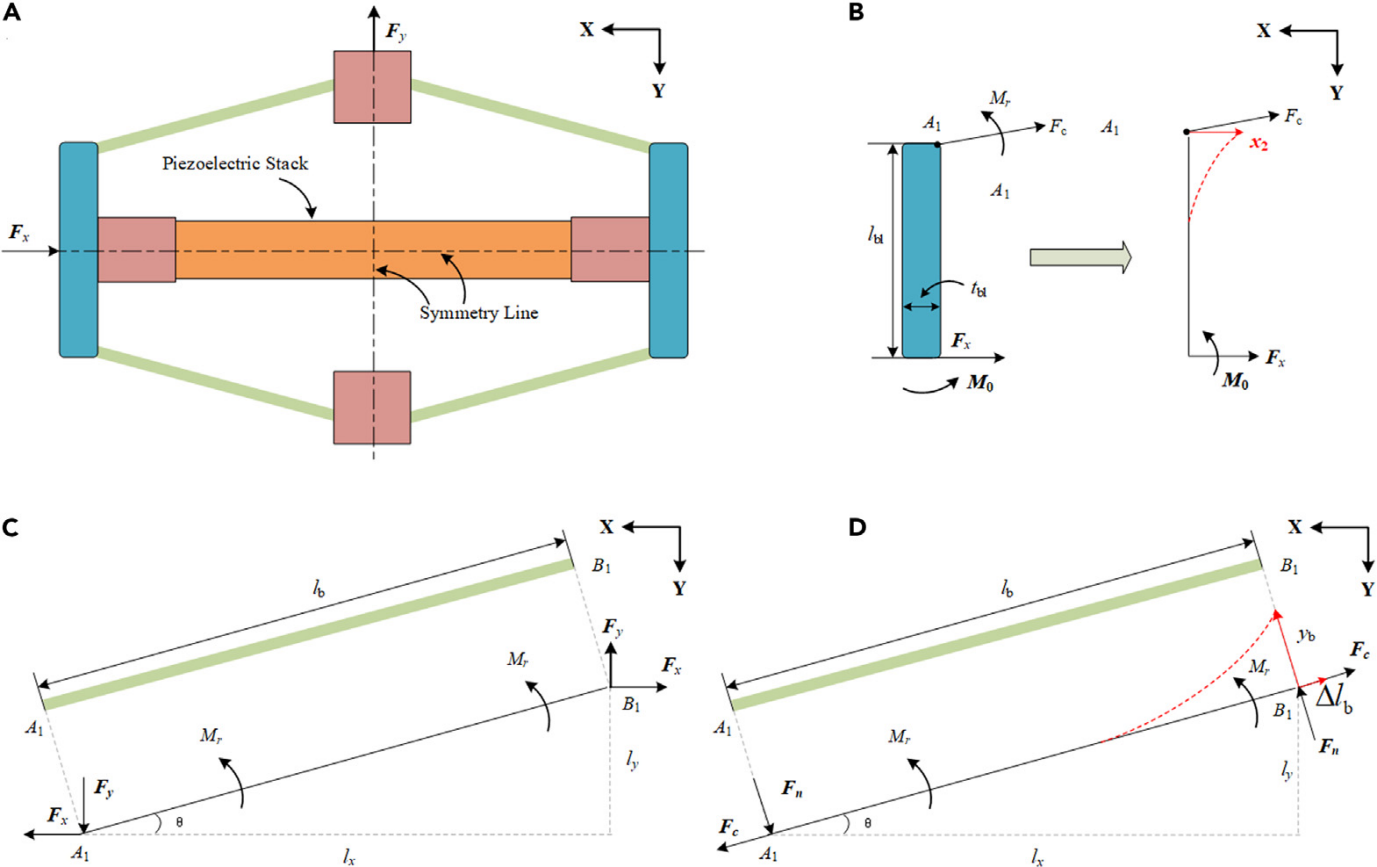
Analysis of a single force amplification frame’s mechanical properties (A) The working principle. (B) The side connecting block. (C) The beam. (D) The beam under equivalent load.

Due to the double symmetry structure in Figure 4A, one amplifier frame’s fourth is explored first. A similar frame thickness is employed for manufacturing and analytical modeling convenience. As illustrated in Figures 4C and 4D, the beam can be simplified into one comparable beam *A*_1_*B*_1_. Figures 4B–4D show the mechanical analysis of the frame. *b* and *t* denote the breadth and thickness of the beam, respectively.

The vertical input force *F*_*y*_ creates the horizontal output force *F*_*x*_, as seen in Figures 4C and 4D. The following equations can be used to compute the normal force *F*_*n*_ and compressive force *F*_*c*_ along the beam direction.

(Equation 1) $$F_{n}=F_{y}\;\cos\;\theta -F_{x}\;\sin\;\theta$$

(Equation 2) $$F_{c}=F_{y}\;\sin\;\theta +F_{x}\;\cos\;\theta$$

where *θ* denotes the beam’s tilt angle with respect to the horizontal direction.

*l*_b_ and *M*_*r*_ are defined as the length and applied moment of beam *A*_1_*B*_1_, respectively. Using the moment equilibrium at point *A*_1_, the following equation can be derived.

(Equation 3) $$2M_{r}=F_{x}l_{y}-F_{y}l_{x}=F_{x}l_{b}\;\sin\;\theta -F_{y}l_{b}\;\cos\;\theta$$

(Equation 4) $$M_{r}=\left(F_{x}l_{b}\;\sin\;\theta -F_{y}l_{b}\;\cos\;\theta \right)/2=-l_{b}F_{n}/2$$

The Euler-Bernoulli beam theory is used to compute the beam’s deformation *y*_b_:

(Equation 5) $$\frac{d^{2}y_{b}}{dl^{2}}=\frac{M\left(l\right)}{E_{f}I_{b}}$$

where *E*_f_ represents the frame material’s Young’s modulus and *I*_b_ = *bt*^3^/12 signifies the beam’s area moment inertia. *M (L)* is also the moment at endpoint *B*_1_, which can be expressed as:

(Equation 6) $$M\left(l\right)=F_{n}l+M_{r},0\leq l\leq l_{b}$$

Referring to Figure 4D, considering that the fixed boundary condition at *A*_1_, the deflection *y*_b_ at *B*_1_ can be determined by inserting Equations 6 into 5 and integrating Equation 5 in light of the boundary condition 0 ≤ *l* ≤ *l*_b_:

(Equation 7) $$y_{b}\left(l_{b}\right)=\frac{F_{n}l_{b}^{3}}{6E_{f}I_{b}}+\frac{M_{r}l_{b}^{2}}{2E_{f}I_{b}}$$

The deformation of the beam can be further expressed in the following way by condensing Equation 7:

(Equation 8) $$y_{b}\left(l_{b}\right)=\frac{-F_{n}l_{b}^{3}}{12E_{f}I_{b}}$$

Similarly, let *l*_bl_ and *I*_bl_ indicate the half-length and area moment inertia of the side block, respectively. The following is the deformation of the side block for the frame:

(Equation 9) $$x_{2}\left(l_{\text{bl}}\right)=\frac{F_{x}l_{\text{bl}}^{3}}{3E_{f}I_{b}}$$

where $I_{\text{bl}}=b_{\text{bl}}t_{\text{bl}}^{3}/12$, *b*_bl_ and *t*_bl_ signify the width and thickness of the side block, respectively.

Furthermore, the beam is compressed as the input force is applied to the frame. The elongation *l*_b_ of the beam becomes:

(Equation 10) $$\Delta l_{b}=\frac{F_{c}l_{b}^{2}}{3E_{f}A_{b}}$$

where *A*_b_ denotes the cross-sectional area of the beam.

The piezoelectric stack’s deflection Δ*x*_pzt_ can be computed as:

(Equation 11) $$\Delta x_{\text{pzt}}=\frac{F_{x}l_{\text{pzt}}^{2}}{E_{\text{pzt}}A_{\text{pzt}}}$$

Finally, because the total deformation along the *x*-axis is zero according to the compatibility theory, the following connection can be obtained:

(Equation 12) $$y_{b}\;\sin\;\theta =\Delta x_{\text{pzt}}+\Delta l_{b}+x_{2}\;\cos\;\theta$$

By substituting Equations (Equation 8), (Equation 9), (Equation 10), (Equation 11) into Equation 12, we may get the subsequent equation.

(Equation 13) $$F_{x}\left(\frac{\sin^{2}\;\theta l_{b}^{3}}{12E_{f}I_{b}}-\frac{l_{\text{pzt}}^{2}}{E_{\text{pzt}}A_{\text{pzt}}}-\frac{l_{\text{bl}}^{3}\;\cos\;\theta }{3E_{f}I_{b}}-\frac{\cos\;\theta l_{b}^{2}}{3E_{f}A_{b}}\right)=F_{y}\left(\frac{\sin\;\theta l_{b}^{2}}{3E_{f}A_{b}}+\frac{\sin\;\theta \;\cos\;\theta l_{b}^{3}}{12E_{f}I_{b}}\right)$$

Equation 13 comprises a limited set of two variables: *F*_*x*_ and *F*_*y*_. Consequently, the force amplification ratio of the amplifier frame (*N* = *F*_*x*_/*F*_*y*_) can be calculated.

(Equation 14) $$N=\frac{F_{x}}{F_{y}}=\frac{\frac{\sin\;\theta l_{b}^{2}}{3E_{f}A_{b}}+\frac{\sin\;\theta \;\cos\;\theta l_{b}^{3}}{12E_{f}I_{b}}}{\frac{\sin^{2}\;\theta l_{b}^{3}}{12E_{f}I_{b}}-\frac{l_{\text{pzt}}^{2}}{E_{\text{pzt}}A_{\text{pzt}}}-\frac{l_{\text{bl}}^{3}\;\cos\;\theta }{3E_{f}I_{b}}-\frac{\cos\;\theta l_{b}^{2}}{3E_{f}A_{b}}}$$

#### Theoretical dynamics model of the piezoelectric stack

The mechanical equation of motion for the simplified single degree of freedom model system is derived from ref.^55^

(Equation 15) $$\ddot{x}\left(t\right)+\omega_{n}^{2}x\left(t\right)-n\omega_{n}^{2}d_{33}V\left(t\right)=\frac{N}{m_{p}}F_{y}\left(t\right)$$

The variables *x*(*t*) and *V*(*t*) represent the displacement and voltage responses, respectively; $\omega_{n}=\sqrt{E_{PZT}A_{pzt}/mL}$ is the natural frequency of a single piezoelectric stack, in which *E*_PZT_ and *d*_33_ are the Young’s modulus and the charge constant of the piezoelectric material. *A*_PZT_, *L,* and *m* are the cross-section area, length, and mass of the piezoelectric stack. *n* is the quantity of piezoelectric layers included in the piezoelectric stack utilized within the harvester.

### The electric equation could be expressed as

(Equation 16) $$\dot{V}\left(t\right)+\frac{V\left(t\right)}{RC_{p}}+\frac{nm\omega_{n}^{2}d_{33}}{C_{p}}\dot{x}\left(t\right)=0$$

where $C_{p}=n\varepsilon_{33}^{T}A_{PZT}/t_{p}$ is the capacitance of the single piezoelectric stack, $\varepsilon_{33}^{T}$ is the dielectric constant of the piezoelectric material measured at constant stress “T.” *t*_p_ is the thickness of the single piezoelectric layer. The instantaneous and average power outputs of the PEH delivered to the external resistor *R*_p_ can be calculated from the voltage response by

(Equation 17) $$P\left(t\right)=\frac{V^{2}\left(t\right)}{R_{p}}$$

(Equation 18) $$\bar{P}\left(t\right)=\frac{1}{T_{t}}\int_{0}^{T_{t}}\frac{V^{2}\left(t\right)}{R_{p}}dt$$

where *T*_t_ is the total time span.

#### Simulation and experimental dynamic responses

The mechanical structure of the MSJ and axle pin was designed using the computer-assisted engineering (CAE) software package Siemens NX. A finite element analysis (FEA) model was established for both the static calculation and dynamical response simulation using the NX Nastran solver. The solution type included SOL 101 linear statistics for the axle pin and SOL 103 response dynamics for the MSJ. The mass block that modulates the resonant frequency was represented in the FEA model by a concentrated mass (i.e., a 0D mesh). The MSJ was represented by an 8-noded three-dimensional hexahedral mesh. RBE2 1D mesh was used to connect the concentrated mass to the top nodes of the MSJ. Figure S1 shows its detailed parameters.

**Figure 5:**
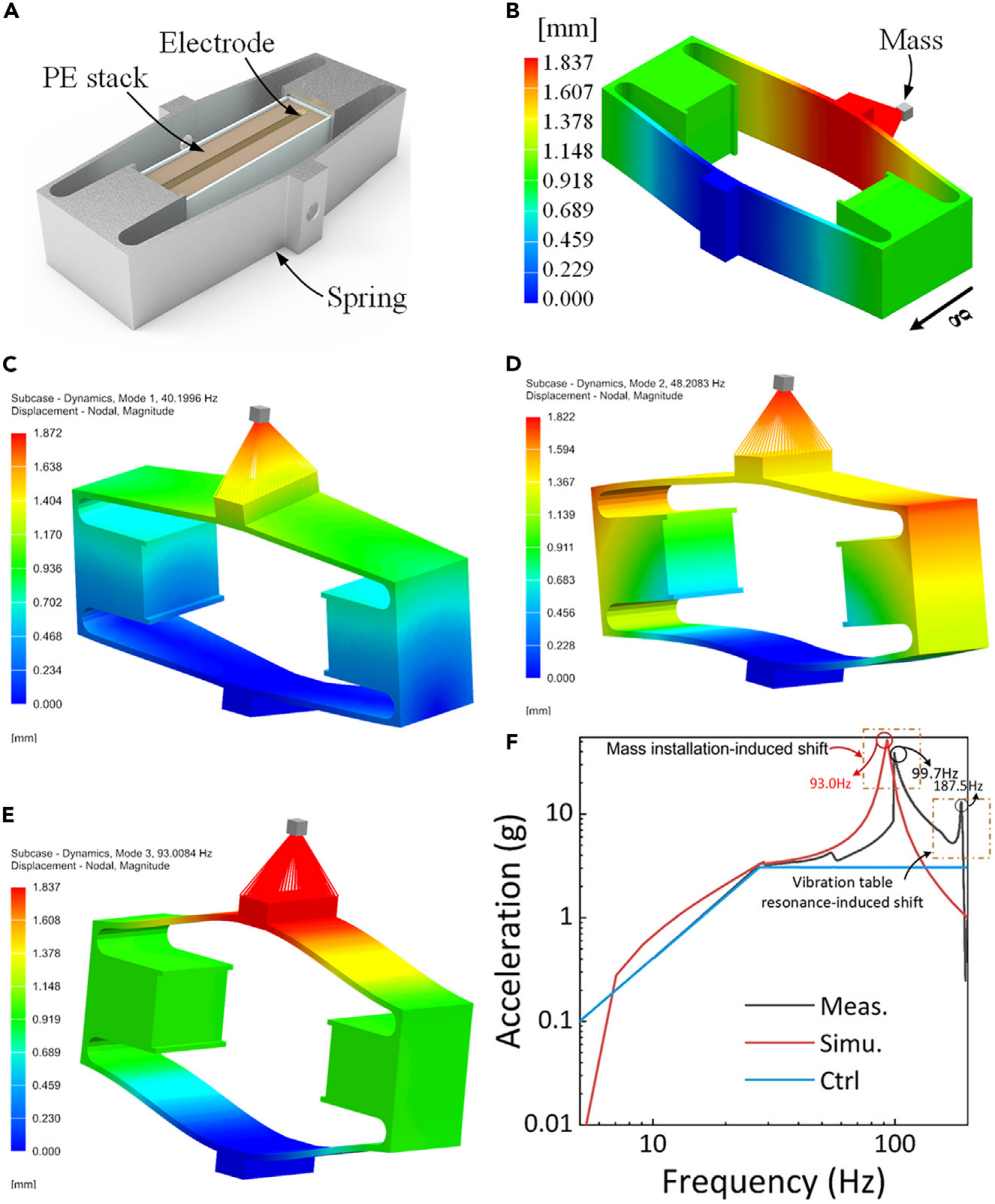
The model and the response characteristics of the MSJ (A) The MSJ with Stacked piezoelectric energy harvesting block. (B) Deformation mapping of the MSJ with resonant frequency adjustment mass on top. (C) The first order modal. (D) The second modal. (E) The third order modal. (F) Vibration excitation and dynamic response curves of the MSJ.

We designed an MSJ (Figure 5A) with two functions: it can convert the vertical vibration acceleration into longitudinal force, thereby driving the piezoelectric (PE) device to transduce energy. However, the four diagonal-stretched thin beam structures of the MSJ can amplify the longitudinal strain (Figure 5B), thereby exciting the PE generator to generate a sufficiently large amount of electricity. Based on the vibration characteristics of the railway track, the resonant frequency point and working bandwidth of the device could be adjusted by configuring the mass unit at the top of the MSJ as shown in Figure 5B, thereby improving the energy conversion efficiency. The response characteristics of the MSJ with additional mass are presented in Figures 5C–5E. The first three order modal frequencies are 40.20, 48.21, and 93.01 Hz, respectively. The vibration excitation and dynamic response were in the frequency range of 5–200 Hz (Figure 5F), which complies with the international vibration test standard.^56^ The blue solid line indicates the vibration acceleration excitation curve defined in the specification and the solid red line indicates the measured vibration response curve at the top of the MSJ. In the measured vibration response curve, three peaks appear in the measurement. Compared with the first three order modal frequencies, they are in one-to-one correspondence. Moreover, we establish a response simulation model for the MSJ. The model can accurately determine the resonant frequency point so that the adaptation design of the PE energy-harvesting device and MSJ can be performed according to the actual railway vibration spectrum.

It is crucial to note that there is a disparity between the simulated and measured responses shown in Figure 5F. Specifically, the resonance frequency from the simulation is 93.01 Hz, which shows a slight deviation from the measured response of 99.7 Hz. In the simulation, the mass concentrated at the top of the MSJ and the fixed constraint at its bottom does not precisely mirror the experimental conditions. The phenomenon of peak value shifting is also evident in both the modeled and experimental results of the MSJ without concentrated mass, as shown in Figure S2. An additional peak in the acceleration response, corresponding to a resonant frequency of 187.5 Hz, is observed. The shift noticed during testing is attributed to the resonance of the vibration table.

### Piezoelectric strain sensing and force magnification by axle-pin

#### Design of the axle pin sensor

**Figure 6:**
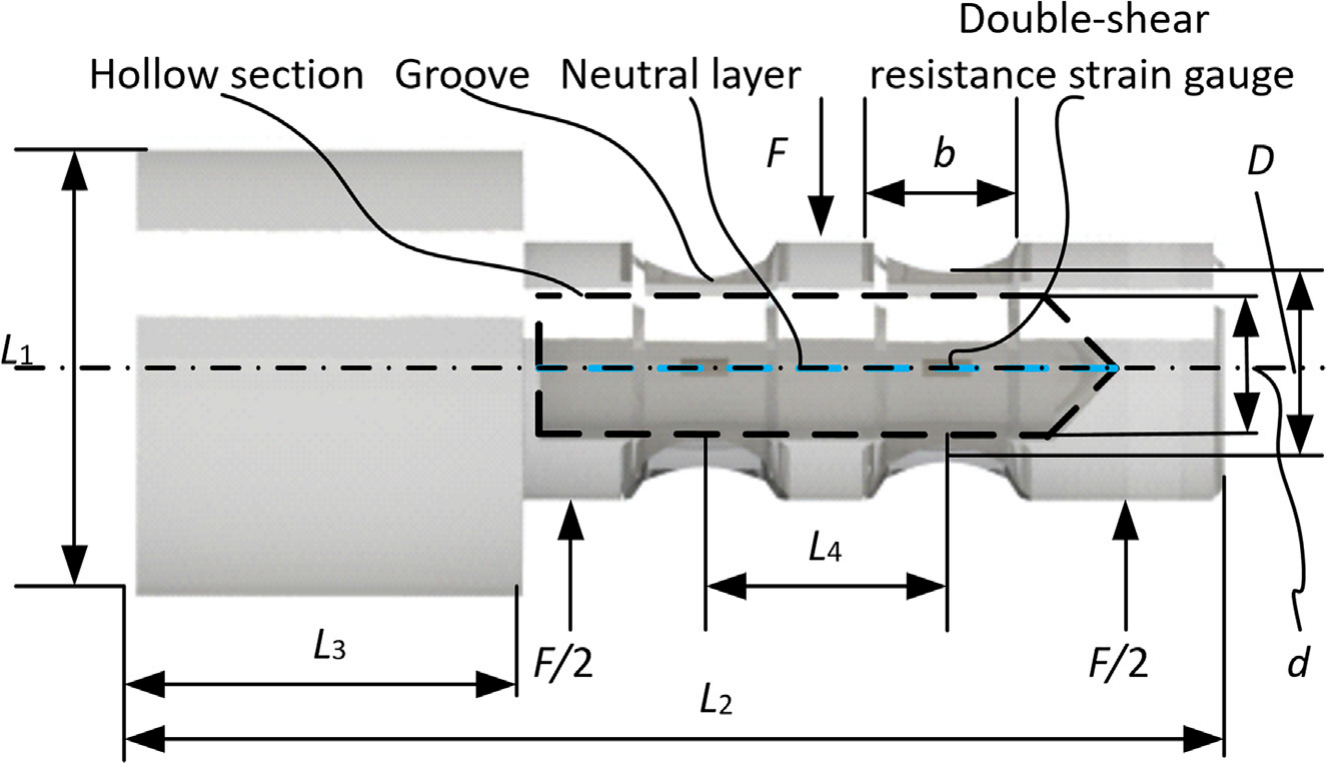
Schematic diagram of the axle pin sensor.

**Figure 7:**
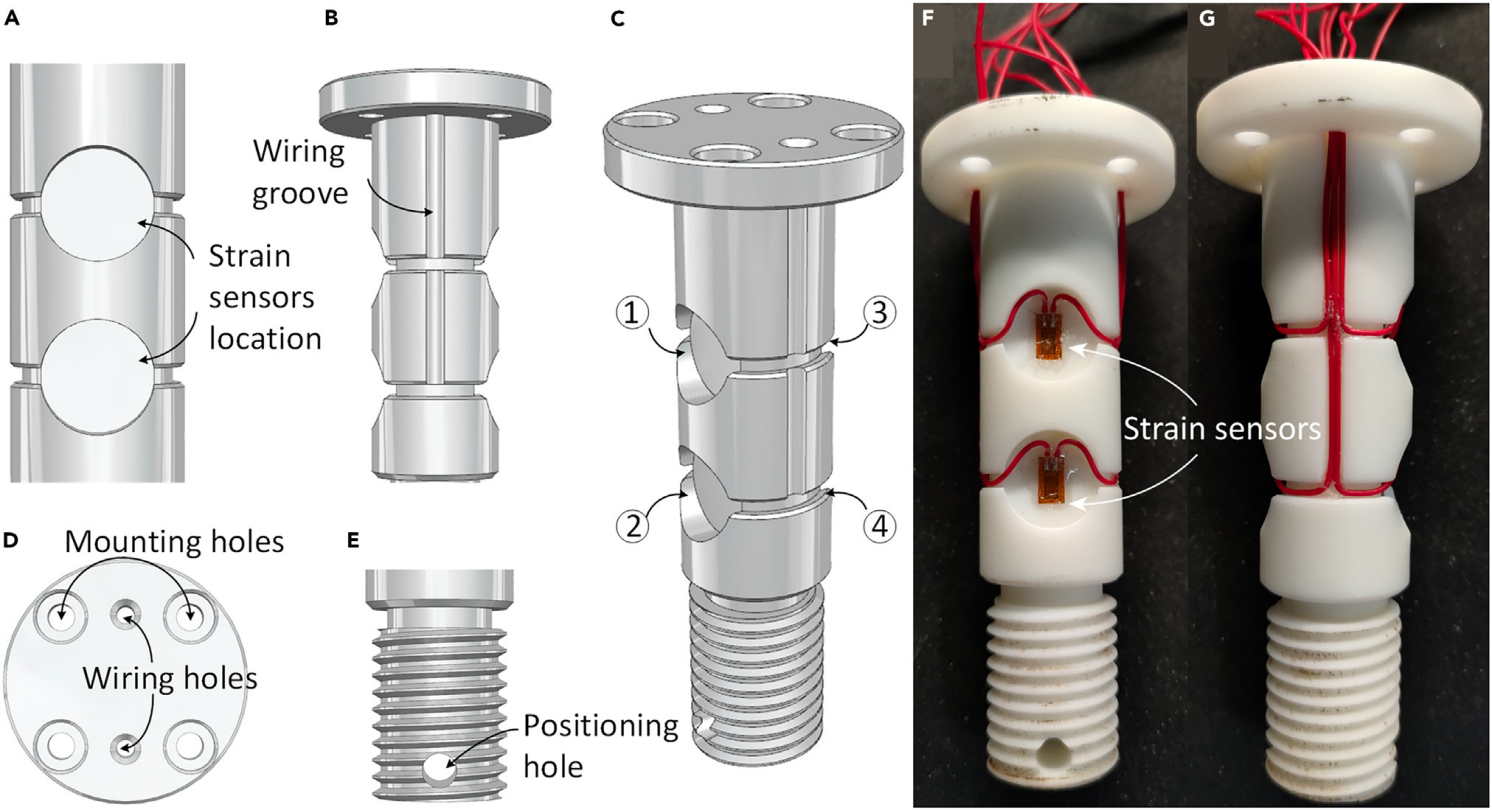
Wiring layout of the axle pin sensor (A) Adhesive locations for strain gauges. (B) Partial views of the wiring groove. (C) Overall views. Numbers indicate where the four strain sensors are attached. (D) Top views of positioning holes and wiring holes. (E) Partial views of the positioning hole. (F) Strain gauges paste diagram. (G) Wiring layout.

To detect the force of the switch machine rod using the strain sensor, it is necessary to amplify the weak voltage signal of the strain sensor and convert it into a force signal. Thus far, we designed a shaft pin structure. The schematic diagram and the stereogram, together with the parameter annotation of the axle pin sensor are shown in Figures 6 and 7, respectively. The axle pin sensor has a cylinder element with a hollow-tube shape and only bears the shear force while working. There are two grooves arranged on the surface of the axle pin sensor symmetrically. Double-shear resistance strain gauges are pasted in the center of the grooves forming a Wheatstone bridge. The working principle is that the external force changes the geometric size of the double-shear piezoresistive strain gauges as well as changes their resistance. The resistance change causes the variation of the output voltage of the Wheatstone bridge. The output voltage of the axle pin sensor is proportional to the strain of the hollow section. and attached four strain sensors to both sides of the bearing area of the shaft pin to form a Wheatstone bridge for sensing the force information of the shaft pin (Figure 7C).

The maximum bending stress *σ*_max_ at the neutral layer of the hollow section is^57^:

(Equation 19) $$\sigma =\frac{\pi D^{3}}{32}{\left[1-{\left(\frac{d}{D}\right)}^{4}\right]}_{\max}$$

where *d* is the width of the hollow section of the neutral layer; and *D* is the minimum diameter of the groove (unit: m).

The maximum bending strain *ε*_max_ is:

(Equation 20) $$\varepsilon =\frac{\sigma_{\max}}{E\frac{8Fb}{\pi D^{3}{\left[1-{\left(\frac{d}{D}\right)}^{4}\right]}_{\max}}}$$

where *F* is the bearing capacity of the pin sensor (unit: N); *E* is the elastic modulus (unit: Pa); and *b* is the width of the groove (unit: m).

### The output sensitivity *S* is

(Equation 21) S=Kε

where *K* is the sensitivity coefficient of the double-shear piezoresistive strain gauge.

Through the analysis of Equations 19 and 20, it can be found that the width, *b,* of the groove of the axle pin sensor has an important influence on the performance of the whole system. It is necessary to ensure that the stress and strain of the neutral layer of the hollow section are the maximum values of the axle pin sensor. The *b* value is two times the base length of the double-shear resistance strain gauge. The minimum outer diameter of the groove of the designed axle pin sensor is *D* = 22 mm. Other parameters are as follows: length *L*_1_ = 38 mm, *L*_2_ = 90 mm, *L*_3_ = 32 mm, *L*_4_ = 20 mm, the width of the hollow section of the neutral layer *d* = 10 mm, the rated load *F* = 10 kN, the material is 40CrNiMoA, and the elastic modulus *E* = 2.1 × 10^4^ kg/mm^2^.

### Modeling and calibration of the axle pin sensor

**Figure 8:**
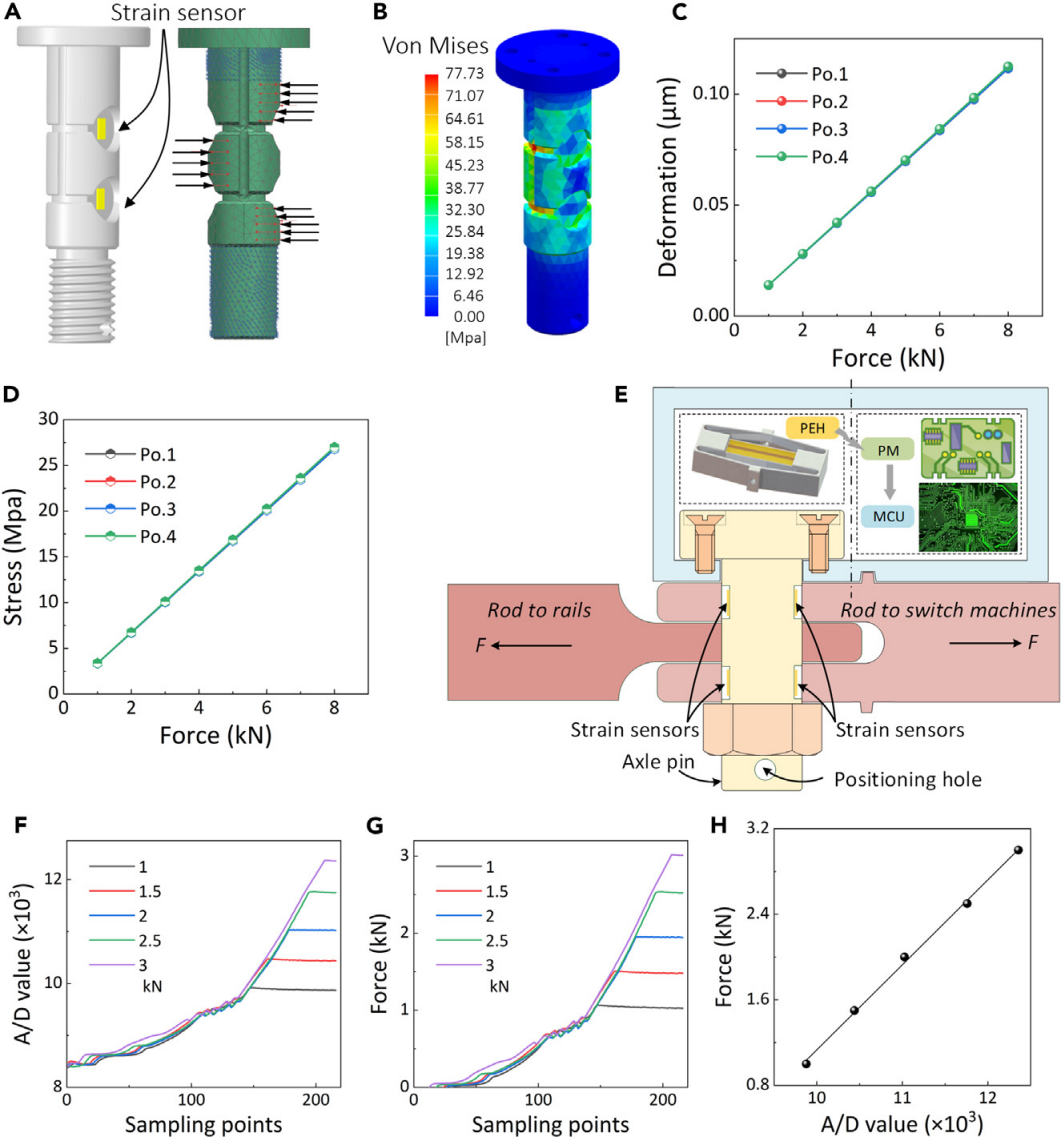
Piezoelectric strain sensing and force magnification by axle-pin (A) Design of the force-sensing axle pin with four as-fabricated strain sensors. (B) Von Mises stress fields of the axle pin under the action of the switching force of the turnout switch machine. (C) Deformation at four placement points of the strain gauges. (D) Stress at four placement points of the strain gauges. (E) Experiment setup for calibration. (F) A/D value output varies with sampling points. (G) Force output varies with sampling points. (H) Calibrated force value with the A/D output of the strain sensor. The axle pin sensor exhibits good linearity.

We designed a shaft pin structure, and attached four strain sensors to both sides of the bearing area of the shaft pin to form a Wheatstone bridge for sensing the force information of the shaft pin as shown in Figure 8A. Finite element analysis was performed to aid the design process and perform a stress-field check (Figure 8B). To enhance the detection accuracy, the absolute values of strain values of the four strain gauges should be the same as much as possible when subjected to the same load, and two strain gauges should be in tension and two in compression. Therefore, four strain gauges were numbered, the load was gradually increased, and the respective stress and strain values were observed and recorded. The deformations and stresses at four strain gauges are described in Figures 8C and 8D, respectively. They are almost the same in the same position. Figure 8E shows the experiment setup for the strain sensor calibration.

A tensile testing machine (Outsmart ZQ-990) was used to calibrate the axle pin sensor and obtain a linear relationship between the A/D value of the strain bridge output and the force on the shaft pin. When converting the tension value, the tension value displayed by the tension machine at a constant force output and stable analogue-to-digital (A/D) value received by the receiver were selected (Figures 8F and 8G), and the least-squares method was employed to determine the conversion relationship between the A/D value and the output value of the tensile machine. The experimental results showed that the shaft pin sensor exhibited good linearity within the detection range (Figure 8H).

### Feature of the strain sensor

Our strain sensor is distinguished by its straightforward installation and robustness, effectively addressing the limitations of conventional strain sensors. Common sensors, typically standalone devices, require attachment to the surfaces under examination and are vulnerable to adverse climatic conditions, which can compromise their accuracy.

To overcome the challenges of size, power requirements, and installation complexity associated with standard commercial strain sensors, particularly for measuring the force on the connecting rod of a switch machine, we have designed a shaft pin construction. This design incorporates four strain sensors on each side of the shaft pin’s bearing area, forming a Wheatstone bridge configuration for efficient force data detection. The axle pin functions as both a structural fastener and a sensor, which simplifies installation and reduces cable usage. We recommend using four strain gauges in this setup, with two under tension and two under compression, to enhance detection accuracy.

Our strain sensor also excels in measuring strain in high-speed railway turnouts under varied and challenging environmental conditions. The sensors are strategically embedded within the connecting rods, offering protection against environmental stressors such as extreme temperatures and moisture, as illustrated in Figure 8E. This arrangement ensures data accuracy and consistency. The superior adhesion of the strain gauge during fabrication enhances the stability of our shaft pin sensor, ensuring reliable performance even under varying vibratory conditions. This robustness addresses a common failure point in traditional sensors, where inadequate bonding during installation leads to inconsistent results.

### On-site system integration testing of the vibration-powered force sensing system in high-speed railway turnout

#### On-site testing producer of the vibration-powered force sensing system

Before initiating on-site testing, it is essential to assemble all individual components into a functional unit, known as the VFSS. The internal structure of this device is depicted in Figure 8E. The connection between the MSJ (Multi-Stability Joint) and the axis pin is secured using a bolt attached to the upper surface. A mass block is firmly affixed to the top of the MSJ for frequency modulation purposes. The piezoelectric stack is incorporated into the MSJ through the application of external stress on its top and bottom surfaces. When this external force is removed, a pre-tightening force is applied to the piezoelectric stack to prevent its downward movement. The connection between the PM (Power Module) and the MCU (Microcontroller Unit) involves attaching two output wires to the PM. The MCU controls the operation of both the strain sensor and the wireless transmission circuit.

The rail vibrations are induced by the interaction between the train wheels and the rail. These vibrations are transmitted to the axis pin through a connecting rod. The MSJ converts the vertical oscillations into horizontal forces exerted on the piezoelectric stack. This stack then generates voltage under the continuous horizontal force stimulation. This voltage is regulated and converted into DC power, which is subsequently used to power the circuits.

For security reasons, the installation of unauthorized equipment by the China Railway Corporation is strictly prohibited, adhering to the guidelines set by the railway department. Therefore, we conducted on-site testing to measure the acceleration on the connecting rod and evaluate the energy harvesting capabilities using a shaker. Additionally, we employed a switch machine test bench to assess the force sensing capabilities and validate its operational effectiveness.

The acceleration test was conducted in the railway turnout section under the jurisdiction of the China High-speed Railway Chengdu Bureau Group. The turnout section used an 80 kg/m steel rail with a No. 18 switch (curvature radius of 1100 m). It is a single-opening switch with a switch crossing (movable-point frog). The opening and closing of the switch rail (i.e., point rails and nose rails) were controlled by the switch machine.

**Figure 9:**
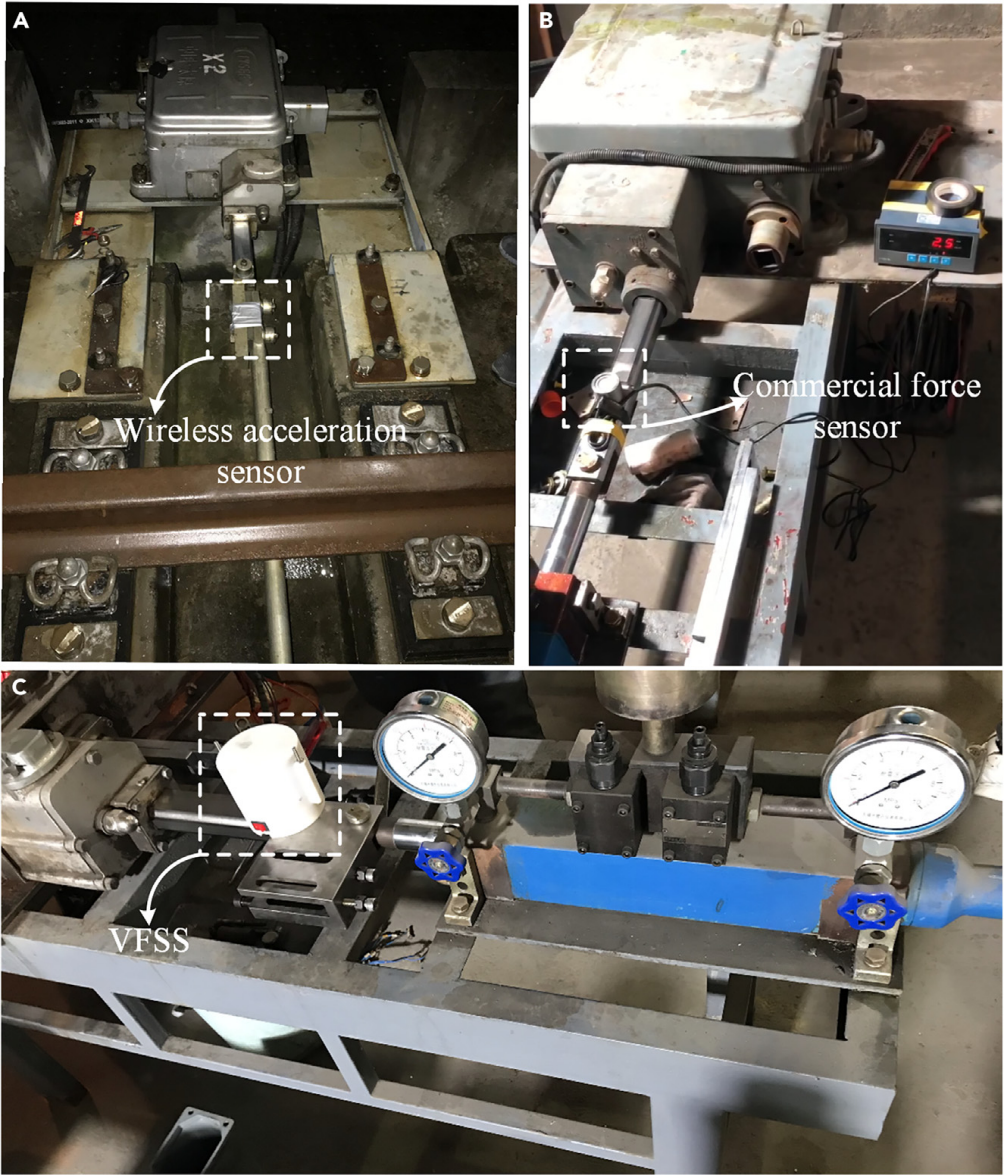
Testing procedure (A) On-site acceleration measurement. (B) Measuring the switching force by the commercial force sensor. (C) Performance validation of the VFSS.

**Figure 10:**
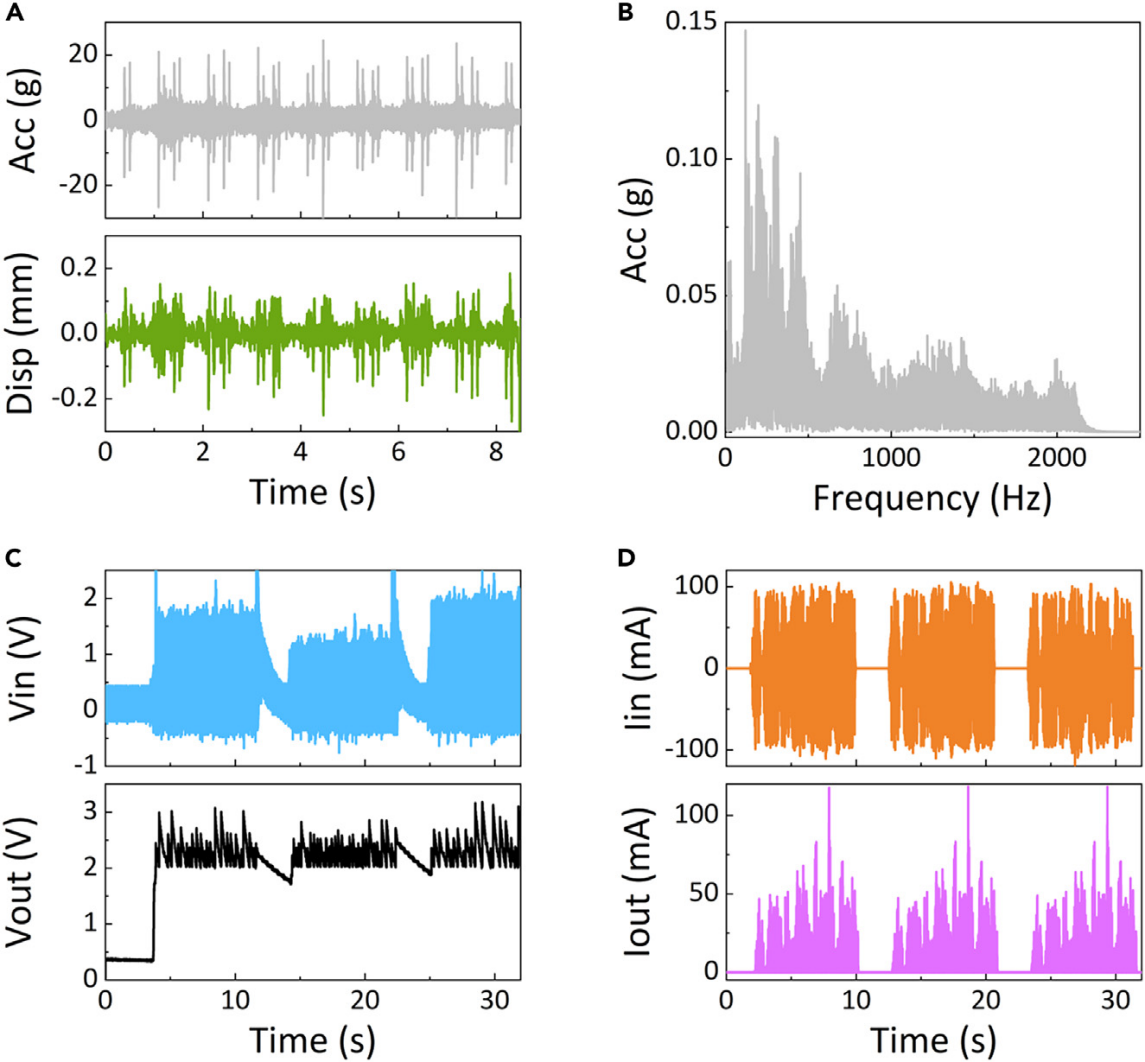
The measured vibration excitation and the power generation capacity (A) On-site measured railway track vibration acceleration and displacement in the turnout section. A set of traveling loads of high-speed railway vehicles. (B) Spectral distribution of the accelerated vibrations of the railway track in the turnout section. (C) Input voltage of the PEH to the PM and the output voltage after the PM under the excitation of the accelerated vibrations of the railway in the turnout section for three sets of traveling loads. (D) Input current of the PEH to the PM and the output current after the PM under the excitation of the accelerated vibrations of the railway in the turnout section. 3 sets of traveling load.

The specific turnout under examination is a sideline, with trains operating at speeds of 80 km per hour. The entire testing procedure is illustrated in Figure 9. We measured the vibration acceleration of the connecting rod using a wireless acceleration sensor during train passages, as shown in Figure 9A. The on-site energy harvesting ability of the Piezoelectric Energy Harvester (PEH) was evaluated by using the recorded acceleration data as input for the shaker’s control software, as depicted in Figure 10. Furthermore, to perform a comparative analysis with the existing strain sensors in the commercial market (shown in Figure 9B), we tested the force sensing capabilities of the VFSS using the well-established switch machine test bench (illustrated in Figure 9C). The force readings obtained from the VFSS demonstrated a high level of consistency with those recorded by the commercially available sensors. This validation process confirmed the accuracy of the VFSS in measuring the force exerted on the connecting rod of the switch machine.

### Power generation capacity of the piezoelectric energy harvester

The experimental setup included a PE vibration energy harvester, electrical circuit module, shake table (THV-2200-3200), control termination, data acquisition unit, and data logger. The measured vibration acceleration data of the railway track were imported using the control software. The PE energy harvester was fixed on a vibration table to realistically simulate the energy-harvesting characteristics under the vibration excitation of an actual railway track. The input and output pads of the electrical circuit module were connected to the energy harvester and data acquisition device, respectively. The data collected by the data acquisition device, namely a digital multimeter (Keithley DMM6500), were then saved to the data logger.

A vibration and shock recorder (S3-E100D40, enDAQ) was utilised to measure the vibration acceleration of the railway track in the turnout section. The onsite measured railway track vibration acceleration and displacement in the turnout section are shown in Figure 10A. This represents one set of traveling loads for a high-speed railway vehicle. The spectral distribution of the vibration acceleration of the railway track in the turnout section is illustrated in Figure 10B. Under the excitation of rail vibration acceleration in the turnout section, the PEH and PM generate the output voltage (Figure 10C) and output electric current (Figure 10D). In total, there were 26 sets of traveling loads (Figure S3). It is evident that the PEH plus PM can stably provide 2–3 V output voltage and an RMS output current of 3 mA, and the RMS output power can reach 6.9 mW, which is sufficient to power the strain sensors, MCU, and wireless transmission to function as a battery-free node for uninterrupted monitoring.

### Power consumption of the vibration-powered force sensing system

To validate the operational capabilities of the VFSS, which includes unattended battery-free sensing and wireless transmission, a test was conducted to measure the power consumption of each sub-system. This test utilized a digital multimeter, specifically the Keithley DMM6500. The strain sensor exhibits a momentary starting current of approximately 73 mA. Upon reaching equilibrium, the peak and average currents are approximately 43 mA and 21 mA, respectively.

### On-site system integration testing

**Figure 11:**
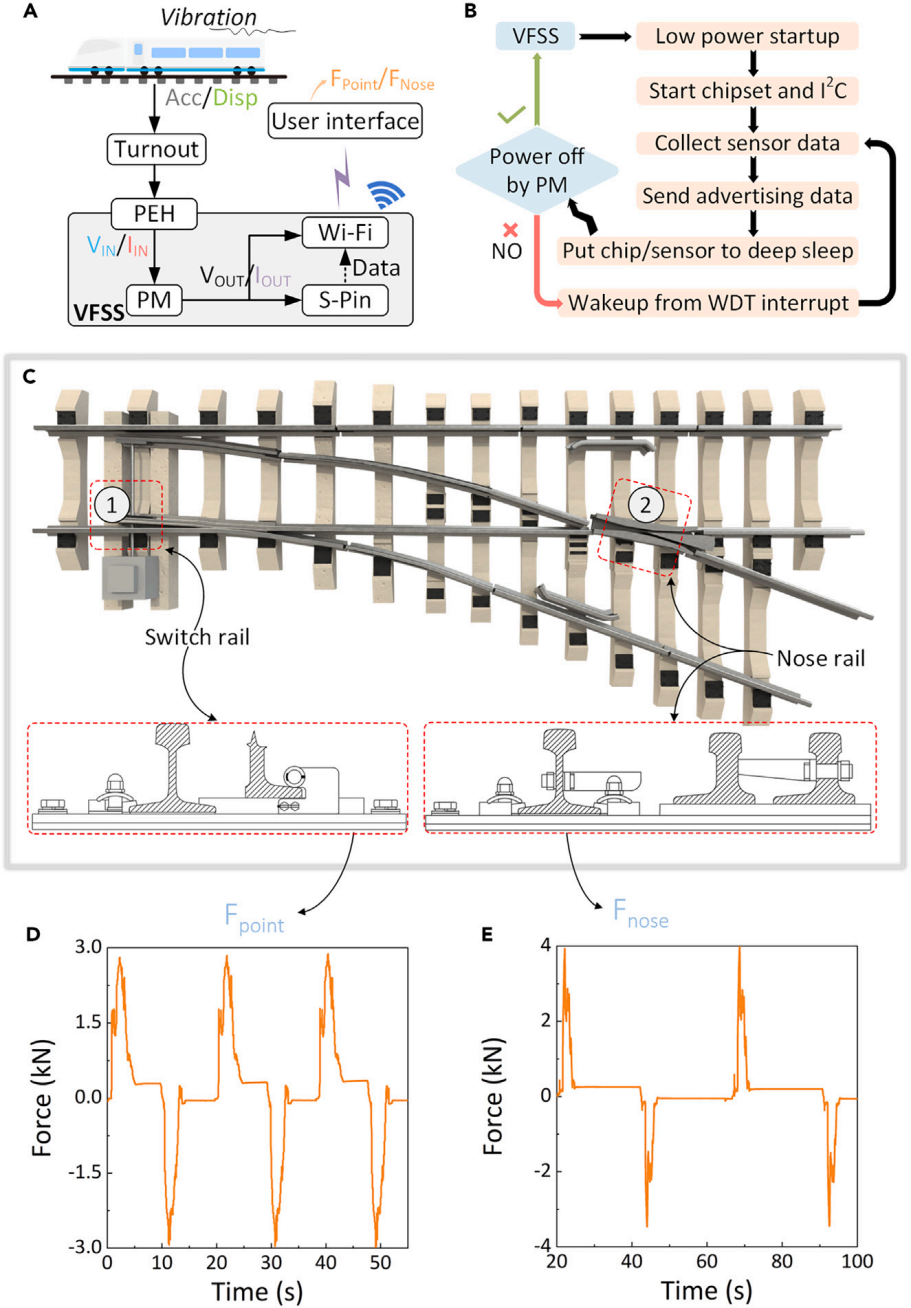
On-site system integration testing of the VFSS in high-speed railway turnout (A) Block diagram of the high-speed railway turnout switching force monitoring system. PEH, Piezoelectric Energy Harvester; PM, Power Management; S-Pin, Sensor Pin; VFSS, Vibration-powered Force Sensing System. (B) Flow chart of low power control and wireless data transmission. (C) Illustration of the measuring location in the high-speed railway turnout section. Measured data of the turnout switching force by the self-powered sensor system: (D) Point rails. (E) Nose rails.

A block diagram of the high-speed railway turnout switching-force monitoring system is presented in Figure 11A. The developed VFSS comprises a PEH, power management (PM) circuit, axle sensor pin (S-Pin), microcontroller (MCU), wireless transmitter, and other peripheral electronic devices along with mechanical components (the schematics are displayed in Figures S4 and S5). A flow chart of the low-loss power control and wireless data transmission processes is shown in Figures 11B. and 11C shows the measuring location in the high-speed railway turnout section. The measured data included the switching force at the point rails (Figure 11D and nose rails (Figure 11E).

Under the broadband (ca. 500 Hz) and small amplitude (i.e., 0.2 mm) dynamic loads of a sixteen-car (25-meter long for one car) trainset rolling stock with a speed of 80 km/h, the VFSS located at the connecting pin of the tie rod of the switch machine can collect an energy of 124.2 mJ (6.9mW∗25m∗16/(80 km/h/3.6) = 124.2 mJ), perceive a conversion force in the range of 0–4 kN, and transmit data wirelessly. The results of this study indicate that the vibration of the rail transit infrastructure can be used as an energy source to construct self-powered wireless monitoring nodes, thus providing an implementable solution for the energy supply of IoT-based microgrids for future green and sustainable transportation.

### Conclusion

We presented a battery-free force sensing axle pin and demonstrated the architecture and functional integration of the system from material preparation, device design, and energy harvesting to power electronics. Under the broadband (ca. 500 Hz) and small amplitude (i.e., 0.2 mm) dynamic loads of a sixteen-car trainset rolling stock with a speed of 80 km/h, the VFSS located at the connecting pin of the tie rod of the switch machine can collect the energy of 124.2 mJ, perceive the conversion force in the range of 0∼4 kN, and transmit data wirelessly. The results of this work indicate that the vibration of the rail transit infrastructure can be used as an energy source to construct self-powered wireless monitoring nodes, thus providing an implementable solution for the energy supply of IoT-based microgrids for future green and sustainable transportation.

### Limitations of the study

The study presents an alternative approach to high-speed railway turnout monitoring using a vibration energy harvesting system based on piezoelectric materials. However, there are limitations to consider. Firstly, the reliance on piezoelectric energy harvesting, particularly with the use of lead zirconate titanate (PZT), raises concerns about material sustainability and environmental impact due to the use of lead. Secondly, the effectiveness of the system is heavily dependent on the frequency and intensity of external vibrations, which might vary significantly in different railway environments, potentially affecting the consistency of energy generation and data monitoring. The system’s durability and long-term performance under varying environmental conditions, such as extreme temperatures and humidity, also remain uncertain. Moreover, the integration of strain film sensors into the bearing shaft pin requires meticulous installation and calibration to ensure accurate data collection, posing challenges in maintenance. Lastly, the cost implications of implementing such a system on a larger scale need further evaluation.

## STAR★Methods

### Key resources table

**Table:** 

REAGENT or RESOURCE	SOURCE	IDENTIFIER
**Chemicals, peptides, and recombinant proteins**		

PZT Powder Source	Baoding Chemicals, China	https://world.taobao.com/
Polyvinyl Alcohol (PVA) Gel	Usolf Chemicals, China	https://world.taobao.com/

**Software and algorithms**		

Finite Element Analysis Software	Siemens NX with NX Nastran solver	https://plm.sw.siemens.com/en-US/nx/

**Other**		

Data Logger	Keithley DMM6500	https://www.tek.com/en/products/keithley/benchtop-digital-multimeter
Tensile Testing Machine	Outsmart ZQ-990	https://world.taobao.com/
Vibration and Shock Recorder	S3-E100D40, enDAQ	https://endaq.com/products/s3-shock-vibration-sensor-s3-e100d40

### Resource availability

#### Lead contact

Mingyuan Gao (goalmychn@gmail.com).

#### Materials availability

The study utilized commercially available PZT powder and standard laboratory materials and chemicals for sample preparation.

#### Data and code availability

Detailed datasets and code that support the findings of this study, along with additional methodological details, are available from the corresponding lead contact upon reasonable request.

### Experimental model and study participant details

#### Experimental platform

The study employed a custom-designed piezoelectric energy harvester and strain sensor system for application in high-speed railway turnouts.

#### Study participant details

Not applicable as this study involves material fabrication and mechanical systems, not human or animal subjects.

### Method details

#### Experimental platform

The piezoelectric energy harvesting block was fabricated using die-pressing and sintering of PZT ceramics, followed by stacking and bonding to create the piezoelectric transducer.

#### Measurement setup

A multi-stability joint (MSJ) was utilized for force magnification and energy transduction, with strain sensors integrated into the bearing shaft pin for force detection.

#### Performance evaluation and comparison

The system was evaluated using a finite element analysis for static and dynamic response simulations, and compared with commercial strain sensors using a switch machine test bench.

### Quantification and statistical analysis

#### Force amplification ratio derivation

Analytical modeling based on literature references was used to predict the force amplification ratio of the MSJ.

#### Theoretical dynamics model

The single degree of freedom model system’s equation of motion was derived and used for system dynamics analysis.

#### Simulation and experimental dynamic responses

Simulations were performed using CAE software, and dynamic response characteristics were validated against on-site measurements using a wireless acceleration sensor and shaker-controlled excitation.

### Additional information

#### On-site system integration testing

The VFSS was assembled and tested on-site at a high-speed railway turnout to evaluate the energy harvesting and force sensing capabilities.

#### Power generation capacity

The power generation capacity of the PEH was assessed using a shake table and data acquisition system to simulate real railway track vibrations.
