# Supplementary material for: Wireless sensing in high-speed railway turnouts with battery-free materials and devices
Source: iScience. 2023 Dec 7;27(1):108663. doi: 10.1016/j.isci.2023.108663 (PMC10753070; doi:10.1016/j.isci.2023.108663)
Supplement: Document S1. Figures S1–S5 [file mmc1.pdf]

iScience, Volume *27*

## Supplemental information

# Wireless sensing in high-speed railway turnouts with battery-free materials and devices

**Yuhua Sun, Yan Yan, Song Tian, Gang Liu, Fei Wu, Ping Wang, and Mingyuan Gao**

# Supporting Information

**This file includes Figures S1-S5.**

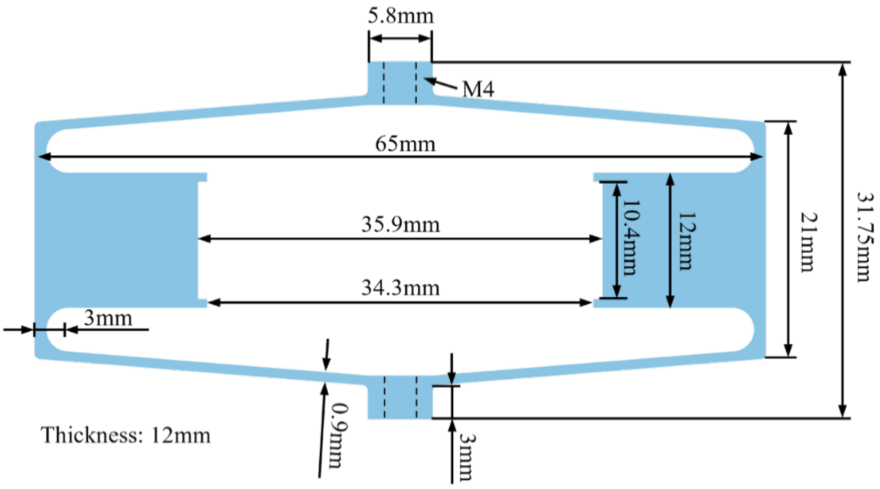


**Figure S1.** Detailed parameters of the MSJ.

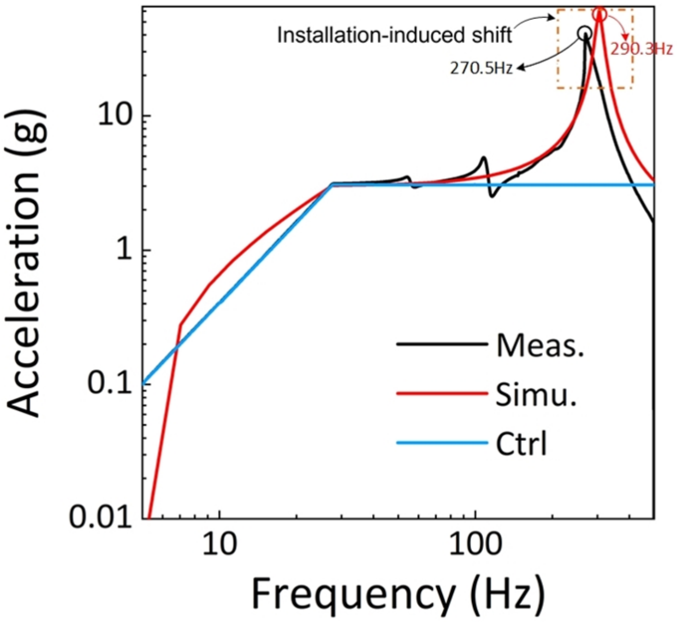


**Figure S2.** Vibration excitation and dynamic response curves of the MSJ without concentrated mass.

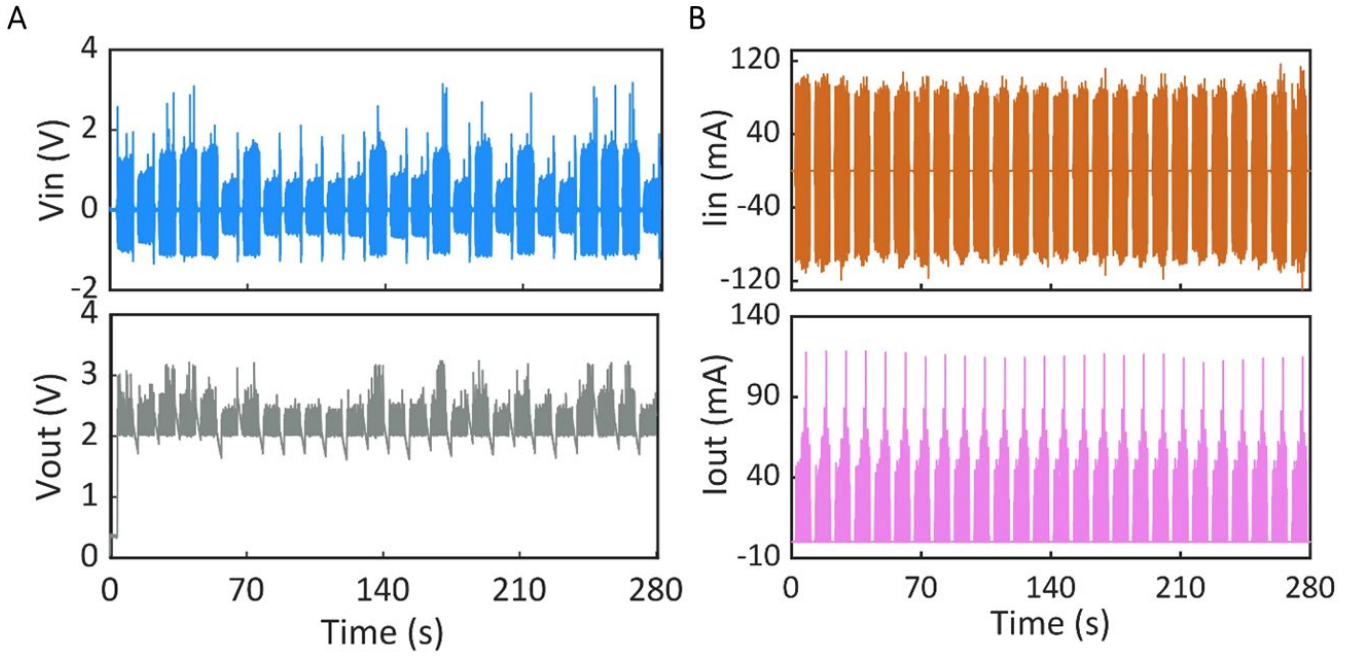


**Figure S3.** Output voltage and electric current of the PEH and PM. (a) Input voltage of the PEH to the PM and the output voltage after the PM under the excitation of the accelerated vibrations of the railway in the turnout section. 26 sets of traveling load. (b) Input current of the PEH to the PM and the output current after the PM under the excitation of the accelerated vibrations of the railway in the turnout section. 26 sets of traveling load.

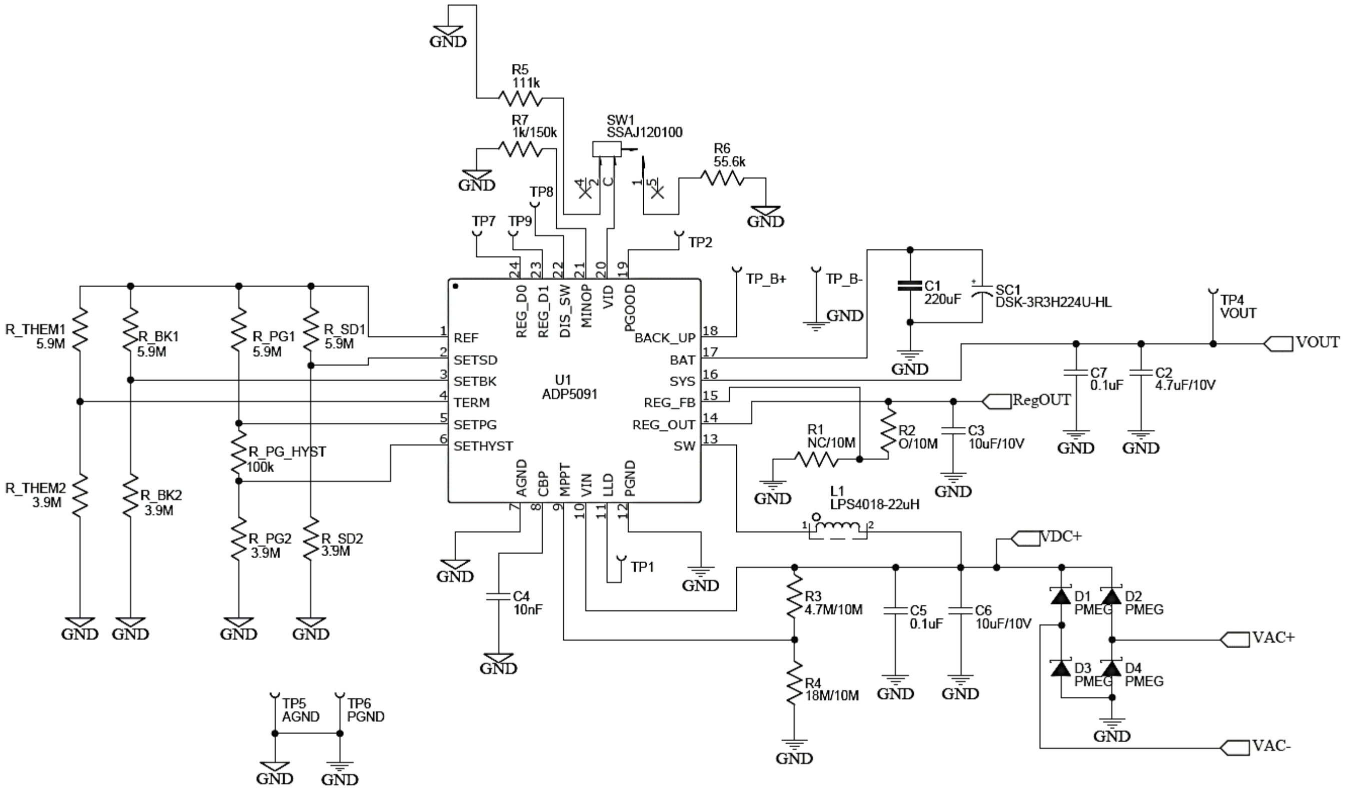


**Figure S4.** Schematic of the PM circuitry.

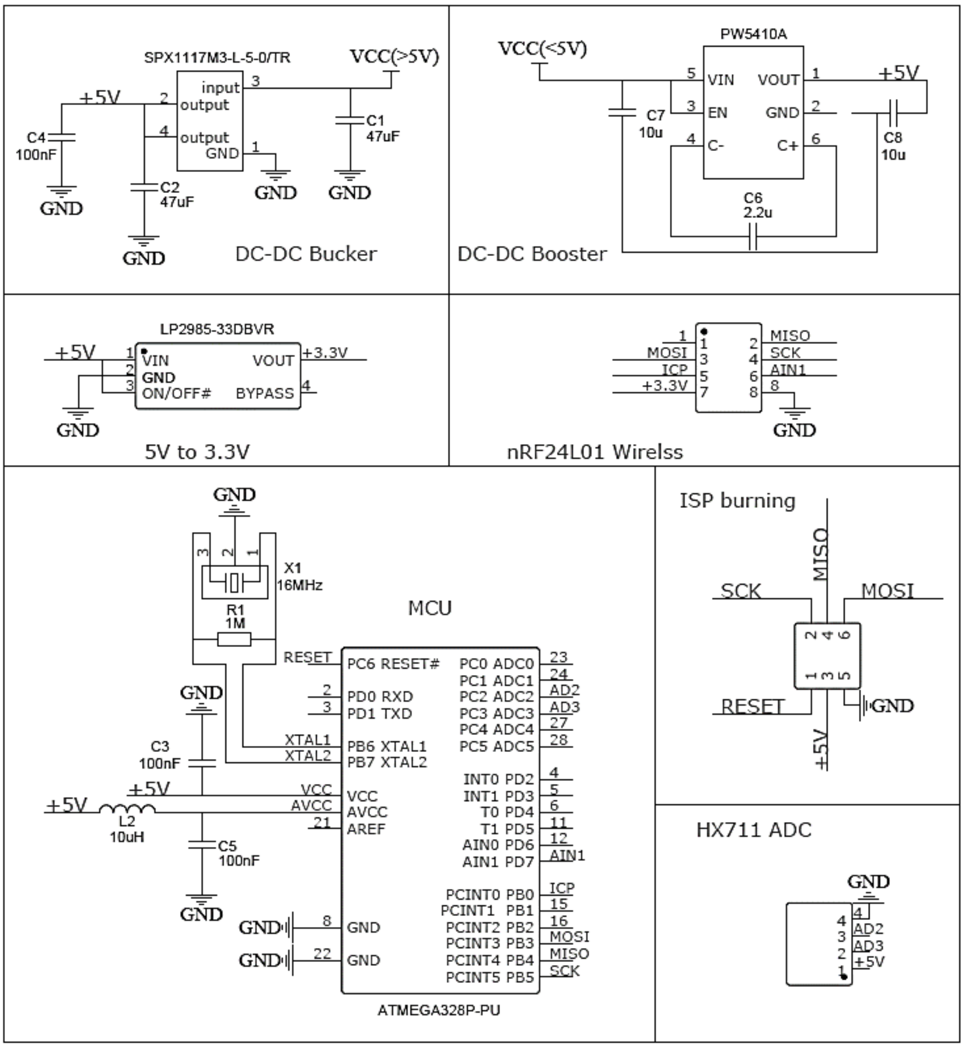


**Figure S5.** Schematic of the ADC, MCU control, and data wireless transmission circuitry.
